# CFD Analysis and Life Cycle Assessment of Continuous Synthesis of Magnetite Nanoparticles Using 2D and 3D Micromixers

**DOI:** 10.3390/mi13060970

**Published:** 2022-06-19

**Authors:** Sergio Leonardo Florez, Ana Lucia Campaña, M. Juliana Noguera, Valentina Quezada, Olga P. Fuentes, Juan C. Cruz, Johann F. Osma

**Affiliations:** 1 Department of Electrical and Electronic Engineering, Universidad de Los Andes, Cra. 1E No. 19a-40, Bogota 111711, Colombia; sl.florez10@uniandes.edu.co (S.L.F.); al.campana10@uniandes.edu.co (A.L.C.); mj.noguera10@uniadnes.edu.co (M.J.N.); op.fuentes@uniandes.edu.co (O.P.F.); 2 Department of Biomedical Engineering, Universidad de Los Andes, Cra. 1E No. 19a-40, Bogota 111711, Colombia; v.quezada@uniandes.edu.co (V.Q.); jc.cruz@uniandes.edu.co (J.C.C.)

**Keywords:** microfluidic systems, iron oxide, magnetic nanoparticles, life cycle assessment

## Abstract

Magnetite nanoparticles (MNPs) have attracted basic and applied research due to their immense potential to enable applications in fields as varied as drug delivery and bioremediation. Conventional synthesis schemes led to wide particle size distributions and inhomogeneous morphologies and crystalline structures. This has been attributed to the inability to control nucleation and growth processes under the conventional conditions of bulk batch processes. Here, we attempted to address these issues by scaling down the synthesis process aided by microfluidic devices, as they provide highly controlled and stable mixing patterns. Accordingly, we proposed three micromixers with different channel configurations, namely, serpentine, triangular, and a 3D arrangement with abrupt changes in fluid direction. The micromixers were first studied in silico, aided by Comsol Multiphysics^®^ to investigate the obtained mixing patterns, and consequently, their potential for controlled growth and the nucleation processes required to form MNPs of uniform size and crystalline structure. The devices were then manufactured using a low-cost approach based on polymethyl methacrylate (PMMA) and laser cutting. Testing the micromixers in the synthesis of MNPs revealed homogeneous morphologies and particle size distributions, and the typical crystalline structure reported previously. A life cycle assessment (LCA) analysis for the devices was conducted in comparison with conventional batch co-precipitation synthesis to investigate the potential impacts on water and energy consumption. The obtained results revealed that such consumptions are higher than those of the conventional process. However, they can be reduced by conducting the synthesis with reused micromixers, as new PMMA is not needed for their assembly prior to operation. We are certain that the proposed approach represents an advantageous alternative to co-precipitation synthesis schemes, in terms of continuous production and more homogeneous physicochemical parameters of interest such as size, morphologies, and crystalline structure. Future work should be directed towards improving the sustainability indicators of the micromixers’ manufacturing process.

## 1. Introduction

Over the past two decades, iron oxide nanoparticles (IONPs) have gained significant attention from academic and industrial researchers, mainly due to their unique properties [1,2]. These properties distinguish them from bulk materials and include small size, strong magnetic responsiveness, large surface to volume ratio, surface reactivity, and ease of functionalization. As a result, they show enhanced reactivity and biological mobility, and superior adsorption capabilities [3]. Consequently, they are well suited for several applications, including biomedical imaging [4,5,6], magnetically-controlled drug delivery [7], tumor hyperthermia [5], bio-sensing [8,9], tissue engineering, cell sorting [10], and the removal of environmental pollutants [2,11].

IONPs can be tailored depending on the final application by adjusting some of their physicochemical properties, including morphology, size, surface functional groups, and magnetization [12,13]. This has been accomplished by the development of different synthesis schemes that intend to control the mechanisms of their assembly, including bottom-up approaches such as co-precipitation of iron salts in an alkaline media [4,11], thermal decomposition of organic precursors [4], hydrothermal growth of crystals [12], microemulsions [12], and microbial methods that involve biomineralization processes [13]. For instance, Barahuie et al. developed a co-precipitation synthesis to obtain IONPs coated by chitosan and phytic acid, for applications in the control of colon cancer cells’ proliferation [14]. Despite the significant progress towards reproducible and scalable synthesis methods, much work is still needed to address challenges in largely heterogeneous particle size distributions, the presence of multiple crystalline phases, alterations in magnetic properties, and variations in morphologies and electrochemical properties [15,16,17,18].

Due to the ease of implementation, co-precipitation schemes appear attractive for a comprehensive scaling-down process to microreaction systems. With this approach, it might be possible to improve the control over key synthesis parameters such as shear rate, the concentration of reagents, reaction time, and temperature. The final goal is to maximize the reaction performance for the one-step IONP synthesis, which translates into higher production efficiencies of materials with high quality standards that can eventually be commercialized [19,20,21,22]. The use of passive micromixers is an alternative to increase reaction performance. Several passive micromixer geometries have been broadly studied due to their efficient mixing quality such as the Tesla valve [23,24,25,26], SAR [27,28,29], curved [30,31], and 3D serpentine [32,33,34,35]. 

The design of such systems can be first explored in silico, with the aid of several methods including rapid and economic CAD prototyping [36,37], hydraulic circuit analyses [38,39], and computational fluid dynamics (CFD) [40,41,42,43,44,45]. CFD simulations of the micromixers allow the rapid design and testing of the performance of multiple prototypes, by identifying the variables that most impact the efficiency and overall performance of the device for a particular task [40,42,43]. This approach requires to the computational domain to be discretized, which can be performed through several methods including the Finite Difference Method (FDM), Finite Volume Method (FVM), and Finite Element Method (FEM) [46]. A popular software package to accomplish this is Comsol Multiphysics^®^, which incorporates several physics that can be coupled to investigate rather complex situations using the FEM. Some authors have conducted multiphysics simulations to optimize the performance of the systems. These improvements vary depending on the type of micromixer but can range from the design of the micromixer [47,48] to the mode of operation [49]. Moreover, this approach allows the integration of governing equations for different phenomena such as fluid mechanics, heat transfer, mass transport, reaction kinetics, electrochemistry, etc. [50,51,52]. This means that the equations governing different physical parameters can be solved simultaneously, allowing the analysis of multiple variables and their possible relationships.

Life cycle assessment (LCA) is an important tool for the analysis of the potential environmental and human health impacts of a product, process, or activity [53]. This methodology has already been successfully applied to several synthesis processes of nanoparticles including magnetite [54], gold [55], and vanadium-titania [56]. In addition, it has been implemented to analyze the potential impact of various wastewater treatment processes [57]. LCA analysis relies on conducting a detailed inventory of inputs and outputs to and from the system under study, including the amount of materials and emissions, and the consumed energy. The corresponding environmental impacts are then estimated by calculating indexes that are normalized to a selected framework for direct comparison with other closely related processes [58]. Conducting an LCA analysis of magnetite nanoparticles production is a suitable route to assure that its large-scale implementation contemplates possible impacts, as the global demand for these nanoparticles is expected to grow considerably within the next few years [59]. 

Therefore, the purpose of this work is to explore the design and manufacturing of three easy-to-assemble and low-cost micromixers, equipped with unique channel geometries for the high-yield synthesis of magnetite nanoparticles with uniform particle size, distribution, and morphology, and in a continuous processing scheme. Additionally, we conducted an LCA analysis of the synthesis enabled by the micromixers in comparison with the traditional batch co-precipitation method. In this regard, the processes were compared in terms of energy and water consumption, to evaluate changes in the potential environmental impacts with respect to the conventional process. This assessment could contribute to an early identification of process hot spots for intervention prior to a definitive scale-up.

## 2. Materials and Methods

### 2.1. Materials

For the manufacture of the micromixer prototypes, polymethyl methacrylate (PMMA) sheets and a methyl-methacrylate adhesive were purchased at a local shop. For the synthesis of magnetite particles, sodium hydroxide (NaOH) (98%) and tetramethylammonium hydroxide (TMAH) (25%) were purchased from Sigma-Aldrich (St. Louis, MO, USA); iron (II) chloride tetrahydrate (98%) (FeCl_2_·4H_2_O) and iron (III) chloride hexahydrate (97%) (FeCl_3_·6H_2_O) were obtained from PanReac AppliChem (Barcelona, Spain).

### 2.2. Microfluidic Geometry Design 

**Figure 1:**
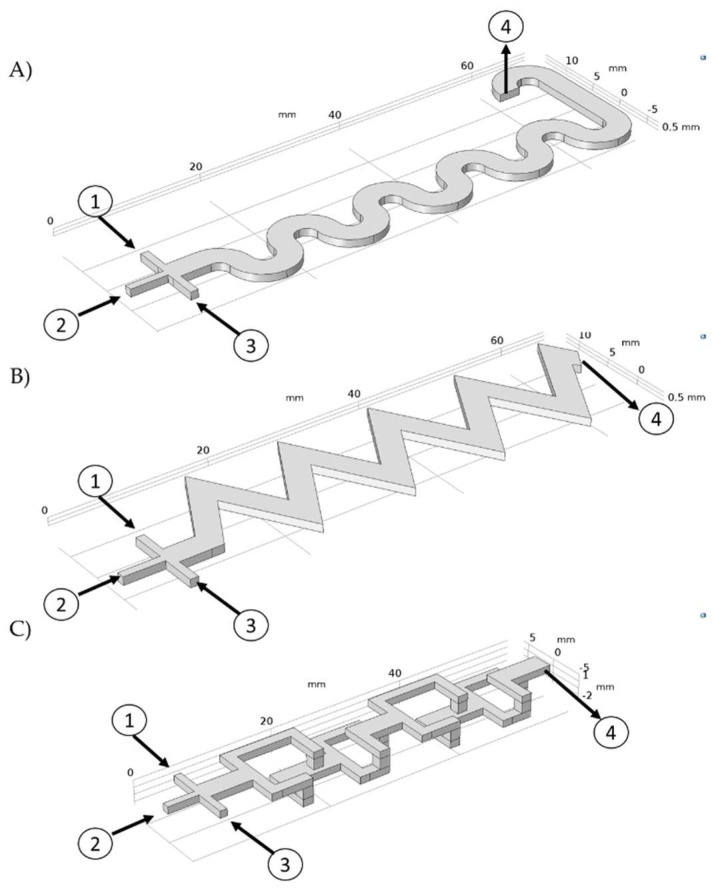
Microfluidic mixer geometries as shown in the Comsol graphics interface. (**A**) Serpentine−based mixer (SB), (**B**) Triangular−based mixer (TB), (**C**) 3D−based mixer (3DB). (1–3. Inflow for FeCl_2_ and FeCl_3_, 2. Inflow for NaOH, and 4. Outflow).

Three microfluidic geometries were proposed and analyzed using Comsol Multiphysics 5.3^®^ (COMSOL, Burlington, MA, USA): a triangular-based mixing pattern (TB), a serpentine-based mixing pattern (SB), and a 3D-based mixing pattern (3DB), which incorporates 90° elbows to abruptly change the fluid directions, achieving a more dynamic mixing (see Figure 1C). This was accomplished by coupling the computational fluid dynamics (CFD), laminar flow, and the chemical reaction engineering and chemical species transport modules of Comsol. All microfluidic systems were designed with the same internal volume (300 mm^3^).

### 2.3. Microfluidic Simulation

To study the behavior of the flow and the reaction, computational fluid dynamics (CFD) simulations were performed in the Comsol Multiphysics^®^ 5.3 software. The reaction conditions were described with the aid of the chemistry module. Equation (1) represents the chemical equation for the formation of magnetite by the co-precipitation method.

(1) $$\mathrm{FeC}l_{2}+2\mathrm{FeC}l_{3}+8\mathrm{NaOH}\to Fe_{3}O_{4}+8\mathrm{NaCl}+4H_{2}O$$

In addition, we coupled the simulation modules of laminar flow and transport of diluted species to describe mixing phenomena inside the micromixers. The laminar flow physic solves the equation of motion for the transport of momentum to generate a velocity profile (Equation (2)).

(2) $$\rho \left[\left(V\cdot \nabla \right)V\right]=-\nabla P+\rho g+\mu \nabla^{2}\;V$$

where *V* is the velocity vector of the fluid, *P* is the pressure, *ρ* is the density of the fluid, *g* is the gravity, and *μ* is the viscosity of the fluid. The transport of species was modeled through Equation (3). The velocity profile calculated from Equation (2) is introduced into Equation (3) and therefore it impacts the mixing dynamics described by the species transport equation.

(3) $$\nabla \left(-D_{i}\nabla c_{i}\;\right)+V\nabla c_{i}=R_{i}$$

where $D_{i}$ is the diffusion coefficient, $c_{i}$ is the concentration of species *i*, and $R_{i}$ is the reaction rate for species *i*.

All the simulation parameters are summarized in Table 1. The three geometries of Figure 1 were subjected to a mesh convergence analysis to determine the minimum number of elements necessary to obtain a stable solution. For this study, five random measurement points were selected along the computational domain and the change in the magnitude of velocity was evaluated as the number of mesh elements increased. As a convergence criterion, it was determined that the velocity magnitude change, obtained with two different meshing levels, should not exceed 3%. An unstructured mesh with free tetrahedral elements was generated. A stationary study was run with the direct solver PARDISO that allows the processes to be parallelized, solving large symmetric or structurally symmetric dispersed linear systems of equations in shared memory multiprocessors [60].

### 2.4. Manufacture of Prototypes

**Figure 2:**
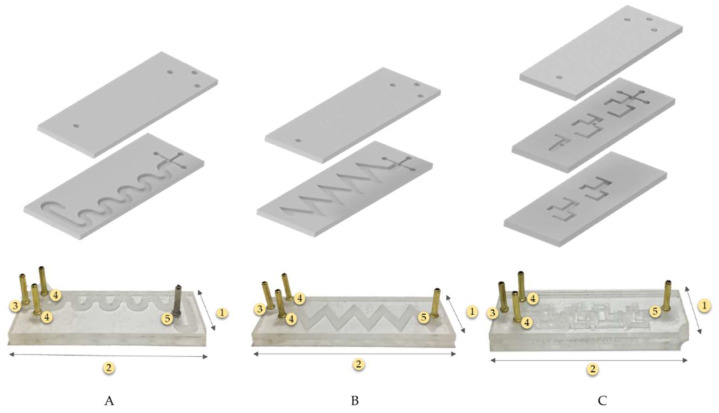
Microfluidic mixers assembly sketch. (**A**) Two layers of the serpentine−based mixer (SB), (**B**) two layers of the triangular−based mixer (TB), and (**C**) three layers of the 3D−based mixer (3DB). (1. Width: 25 mm, 2. Length: 75 mm, 3. NaOH solution Input, 4. Iron chloride mixed solution Input, 5. Output).

Device prototyping was conducted on polymethyl methacrylate (PMMA) sheets of 2.5 mm thickness, using a Speedy 100, 60 W (TROTEC, Germany) laser cutter. The microchannel of the TB and SB micromixers was engraved in the first layer of the substrate and a second layer of PMMA was used to seal the microchannel. Methyl methacrylate was spread on the second layer and both surfaces were then glued together at room temperature (Figure 2A,B). 

For 3DB micromixers, three layers of PMMA were used (Figure 2C). In this case, the microfluidic channels were cut through on layers 1 and 2 of the system via laser cutting and, as shown in Figure 2C, all the system layers were manually aligned and sealed layer by layer with the methyl methacrylate adhesive, similar to the other devices.

Three prototypes were assembled for each configuration (Figure 2). Before running the synthesis reactions, the correct functionality of the assembled prototypes was checked with a continuous water flow to identify possible blockages or leaks.

### 2.5. Synthesis and Characterization of Magnetite Particles

Prior to the nanoparticles’ (NPs’) synthesis, 30 mL NaOH (800 mM) aqueous solution was prepared along with 10 mL TMAH (2% *v*/*v*) aqueous solution, 30 mL ${\mathrm{FeCl}}_{2}\cdot 4H_{2}O$ (100 mM) aqueous solution, and 30 mL ${\mathrm{FeCl}}_{3}\cdot 6H_{2}O$ (200 mM) aqueous solution. The iron chloride solutions were homogenized by magnetic stirring. The Fe_3_O_4_ NPs were manufactured by in situ co-precipitation of the chlorides as they transited through the micromixer. The reaction occurred by injecting 3 mL of the NaOH solution and 6 mL iron chloride mixture solution into the system at a flow rate of 1 mL/min and 0.5 mL/min, respectively (see Figure 2). The produced nanoparticle solutions were kept on 3 mL TMAH (2% *v*/*v*) aqueous solution overnight to avoid agglomeration. All the experiments were carried out in triplicate.

Dynamic Light Scattering (DLS) analysis with a Zetasizer Nano ZS (Zeta-Sizer Nano-ZS, Malvern Instruments, Malvern, UK) was used to determine the average size distribution of the resulting samples in triplicate. Magnetite nanoparticles size, composition, and morphology were examined by high resolution transmission electron microscopy (HRTEM), using a Tecnai F20 Super Twin TMP TEM (FEI company, Hillsboro, OR, USA), operating in bright field at 200 kV. A sample of the NPs synthesized in each micromixer was diluted 1/100 in Milli Q H_2_O, sonicated, and then dropped on a carbon-coated copper grid (Lacey carbon film). Before samples analysis, the NPs were left at 40 °C until dry and then they were cooled down at room temperature. In addition to NP images, electron diffraction in converging beams and elemental analysis via EDS were obtained. Additionally, effective synthesis was confirmed via Fourier Transform Infrared Spectroscopy (FTIR) by a Bruker Alpha II FTIR Eco-ATR (Bruker, Billerica, MA, USA). Spectra were recorded in the range of 4000–500 cm^−1^ with a spectral resolution of 2 cm^−1^. Finally, the purity of the crystalline phase of magnetite was verified via X-ray Diffraction (XRD) (Siemens, Washington, DC, USA).

### 2.6. Life Cycle Assessment (LCA) of the Magnetite (Fe_3_O_4_) Synthesis by Co-Precipitation and Micromixers Methods

#### 2.6.1. Goal and Scope Definition 

The main aim of this LCA analysis was to evaluate the possible environmental impacts of both conventional batch and micromixer-based Fe_3_O_4_ NP synthesis methods. The analysis required a detailed inventory that included the chemicals used, the energy required, and the waste generated. This assessment was based on an attributional approach or descriptive “cradle to door” of laboratory-scale processes. 

**Figure 3:**
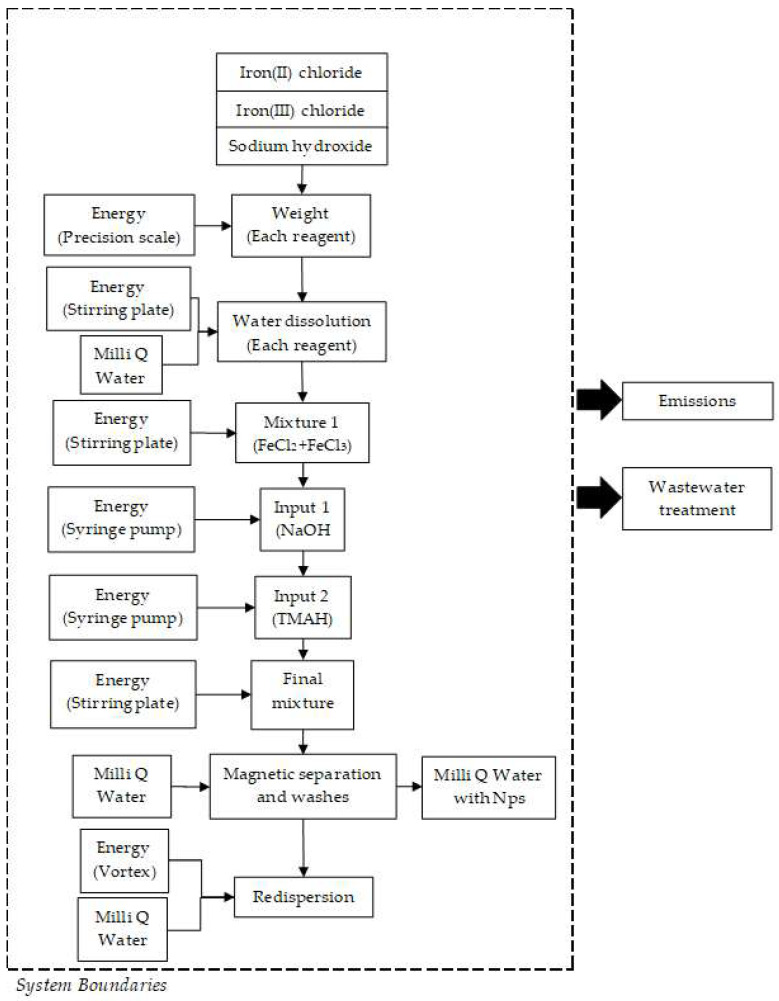
Flowchart diagram of the co−precipitation method for the synthesis of Fe_3_O_4_ NPs. The system boundaries are defined by the dotted framework.

**Figure 4:**
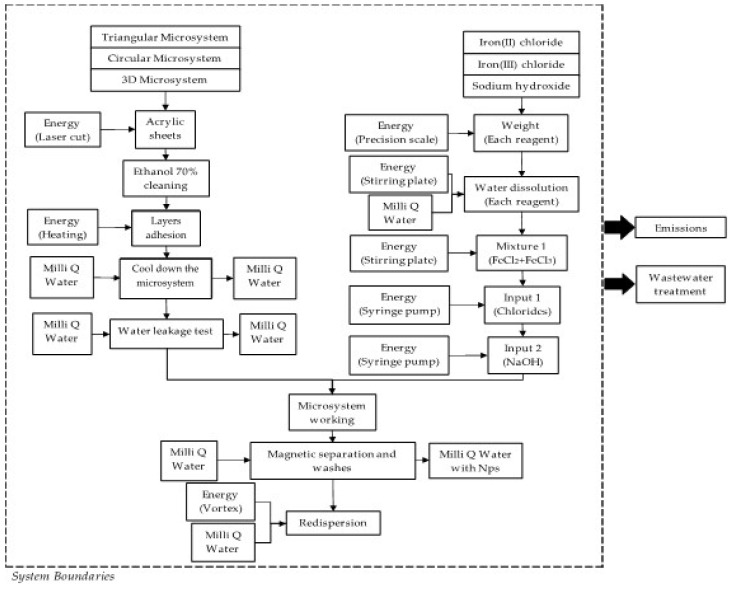
Flowchart diagram of the micromixer−based method for the synthesis of Fe_3_O_4_ NPs. The system boundaries are defined by the dotted framework.

According to the experimental conditions, the functional unit for this analysis was defined as 0.5 g of the NPs produced per batch in each method. However, this functional unit is only valid for the purpose of this work, because the selection of a weight-based functional unit makes sense when comparing production processes to produce an equivalent amount of nanomaterial [61]. Additionally, the system boundaries were defined from the very first input of raw materials until the final Fe_3_O_4_ NP product. Moreover, our approach only contemplated the water and energy consumption of co-precipitation and micromixer-based methods. Figure 3 and Figure 4 show the flowchart diagrams for each of the NP synthesis methods. According to the defined systems boundaries, both emissions and wastewater treatment assessment were the main outputs of the analysis.

#### 2.6.2. Life Cycle Inventory

Data collection in an LCA study consists of determining the relevant reagents, emissions, and waste flows, as well as the energy consumption processes. For each of the NP production processes, the inventory report included (when possible) some experimentally determined data, which were complemented by references to peer-reviewed articles, user manuals, and technical reports and protocols. Importantly, the inventory report of NP synthesis methods was based mostly on laboratory-scale experiments. Table 2 shows the inventory report of raw materials, measurement equipment, water consumption, and energy required for the synthesis of NPs through both the co-precipitation and micromixer-based methods.

## 3. Results

### 3.1. Microfluidic CFD Simulation

Figures S1–S3 in the supplementary information show the mesh convergence analysis for SB, TB, and 3DB, respectively. In the case of SB, convergence was achieved with 300,000 mesh elements. For TB, this was achieved with 200,000 mesh elements, while for 3DB, 400,000 were required. All simulations were performed on a computer equipped with an AMD Ryzen 5 2100 Mhz processor, 4-core, and 16 GB of RAM.

**Figure 5:**
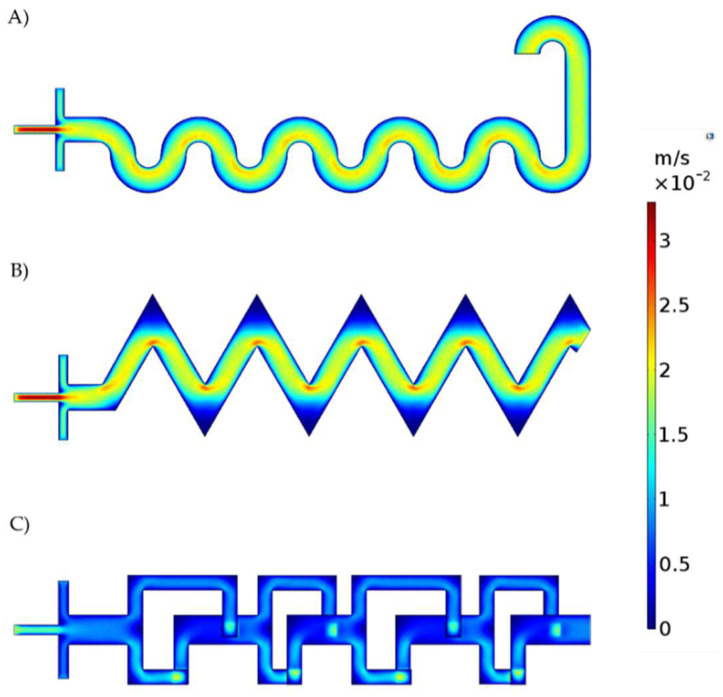
Velocity profile for each micromixer. (**A**) Serpentine−based (SB) micromixer, (**B**) Triangular−based micromixer (TB), and (**C**) 3D−based micromixer (3DB). Apparent flow discontinuities in 3DB are due to the projection of out−of−the plane channel segments.

Although all the input flow rates were the same, different velocity and shear rate profiles developed inside the three microchannel types due to the marked differences in geometry. Figure 5 show the velocity profile for each micromixer. The SB micromixer shows no noticeable zones of dead volume. However, the TB and 3DB micromixers have zones of dead volumes along the sharp corners of the geometries. This could be due to low pressure in such areas of the micromixer where reagents or air bubbles are likely to accumulate [62,63]. The SB and TB micromixers achieved higher maximum velocities compared to 3DB. Moreover, TB developed a velocity profile that resembles that of the SB system. This is most likely due to the sinusoidal flow pattern achieved by this system (Figure 5B). In relation to the generation of the dead zones described above, the fluid tends to move to areas with less hydrodynamic resistance, generating the flow pattern shown in Figure 5.

**Figure 6:**
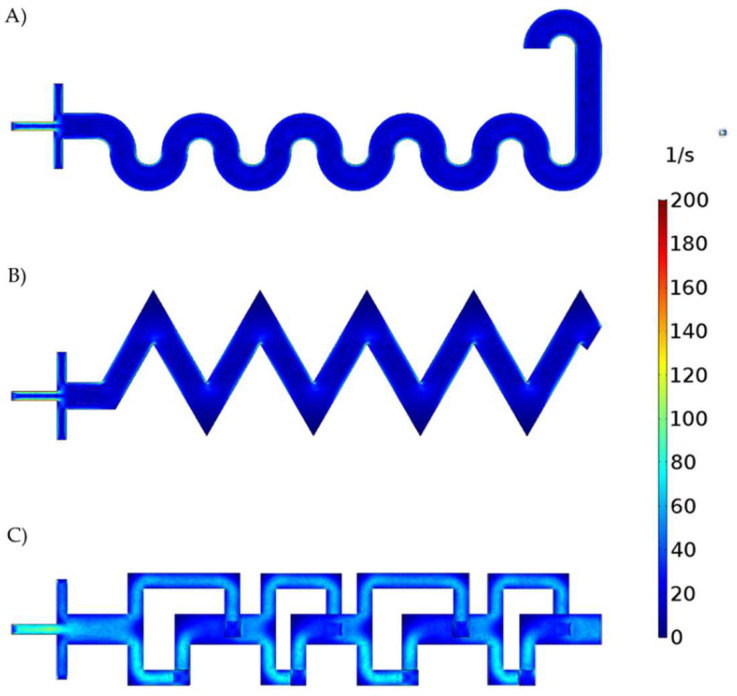
Shear rate for each micromixer. (**A**) Serpentine−based (SB) micromixer, (**B**) Triangula−based (TB) micromixer, and (**C**) 3D−based (3DB) micromixer.

Figure 6 shows the shear rates for each micromixer. The SB and TB developed low shear rate values. In contrast, for the 3DB micromixer, the shear rate reached values as high as 80 s^−1^. This is most likely due to the narrower channels and the abrupt direction changes observed for the reaction mixture in this case. Different levels of shear rate have been reported to strongly impact the NPs’ nucleation and growth processes [64,65].

### 3.2. Reaction Simulation

**Figure 7:**
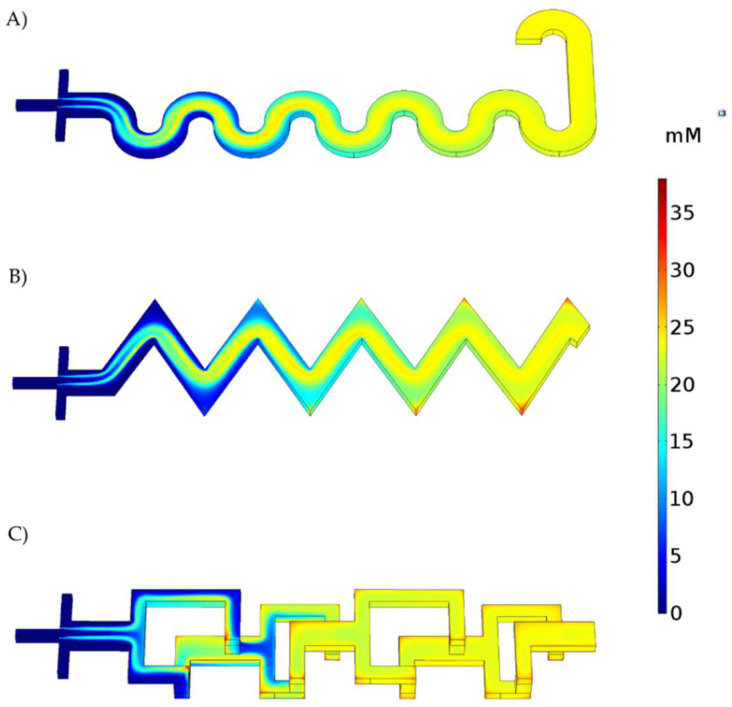
Fe_3_O_4_ NP concentration profiles during transit along each micromixer. (**A**) Serpentine−based (SB) micromixer, (**B**) Triangular−based (TB) micromixer, and (**C**) 3D−based (3DB) micromixer.

Figure 7 shows the concentration distribution of NPs along the microchannels. NPs tend to accumulate in the corners of the TB and 3D geometries. These zones match those of the previously identified dead volumes, which can be seen in Figure 5. The TB system exhibits the highest accumulation level of NPs in the corners, which can be attributed to inefficient mixture of reagents. The SB system shows more efficient mixing, as evidenced by the absence of dead volumes. Finally, although the 3DB system has several dead volume zones, the abrupt changes in the flow direction appear to promote a better interaction between the reacting species. It is important to note that all three configurations arrive at similar concentrations of NPs (i.e., SB = 23.22 mM, TB = 22.43 mM, and 3DB = 23.18 mM). This suggests that the flow rates, dimensions, and channel features are adequate to produce the magnetite NPs.

### 3.3. Synthesis of Magnetite Particles and Characterization of Magnetic Particles

**Figure 8:**
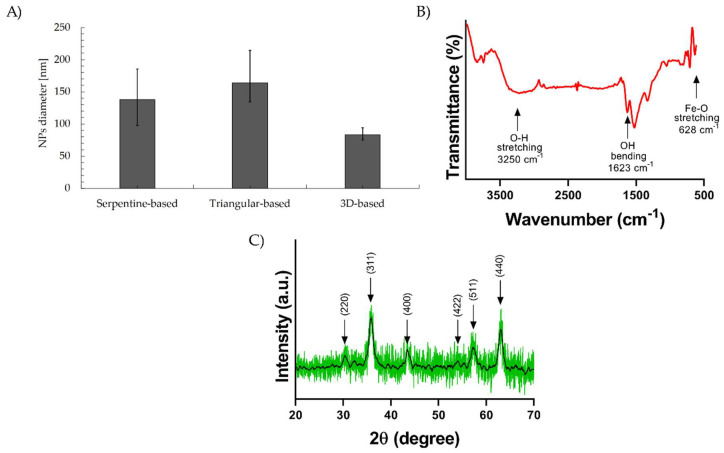
Magnetic nanoparticle characterizations. (**A**) Average diameter of the magnetite nanoparticles produced by each micromixer. For the serpentine−based (SB) micromixer, it approached 138.00 ± 44.00 nm, for the triangular−based (TB) micromixer, it was 163.57 ± 40.15 nm, and finally, for the 3D−based (3DB) micromixer, it was 83.03 ± 9.52 nm. (**B**) FTIR of representative spectra of NPs. (**C**) XRD representative diffractogram of the NPs.

**Figure 9:**
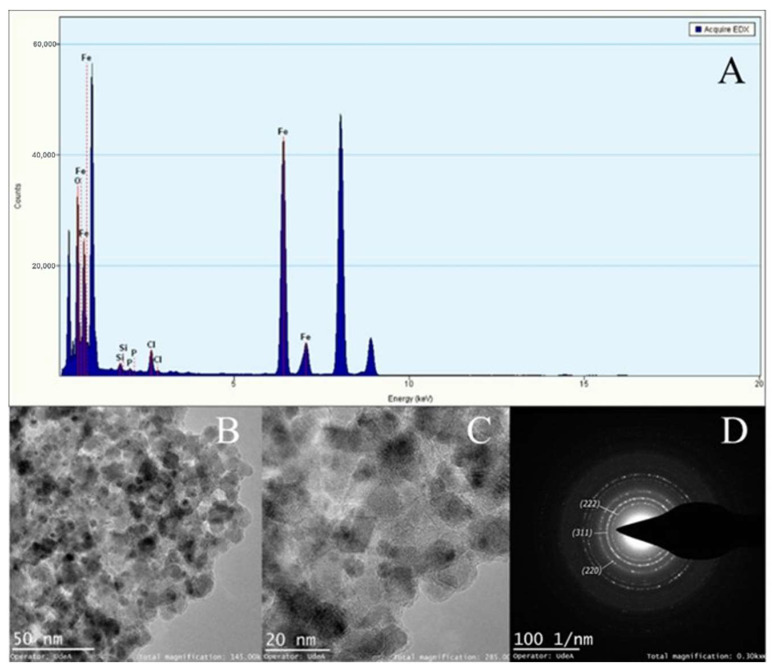
TEM−EDX analysis of magnetite particles manufactured by the SB micromixer (**A**). Elemental analysis confirmed the abundance of iron. (**B**) Low magnification imaging confirmed the typical morphology of magnetite crystals. (**C**) High magnification imaging allowed the calculation of an average particle diameter of 10.14 ± 2.8 nm. (**D**) Selected area electron diffraction with indexed planes that correspond to magnetite crystalline planes.

After NP synthesis, the average particle diameters for the SB, TB, and 3DB micromixers were 401.97 ± 92.70 nm, 259.97 ± 22.45 nm, and 325.33 ± 65.85 nm, respectively. To reduce particle agglomeration, NP solutions were further dispersed with a tip-probe sonicator, Vibra-Cell™ Ultrasonic Liquid Processor (Sonics & Materials, Newtown, CT, USA), and an ultrasonic bath, Branson 5800, 2.5-gallon, 40 kHz (Emerson, St. Louis, MO, USA). DLS analysis for average particle diameters after homogenization are shown on Figure 8A. The SB micromixer presents a 138.00 ± 44.00 nm particle diameter, the TB micromixer 163.57 ± 40.15 nm, and the 3DB device presents an 83.03 nm ± 9.52 nm particle diameter. These results can be explained by the low velocity zones in the SB and TB micromixers, which produce a higher polydispersity than the 3DB micromixer, and thus their larger size distribution [66,67]. Although particles’ average size distribution was reduced for all the NP samples after ultrasonication [68,69], this resuspension method has a limited dispersion capacity for magnetic NPs due to their interactions, and thus, the reported hydrodynamic diameters are still related to NP clusters, as it was confirmed via TEM observation (Figure 9). 

FTIR representative spectra (Figure 8B) exhibit the presence of a band at around 628 cm^−1^, attributed to the stretching vibration mode associated with the metal–oxygen Fe-O bonds in the crystalline lattice of Fe_3_O_4_, which confirms the formation of magnetic nanoparticles. A band at 1623 cm^−1^ and the broad band centered at 3250 cm^−1^ are related to the presence of hydroxyl groups and attributed to OH-bending and OH-stretching, respectively [70]. There was no evidence of PMMA characteristic peaks, from which it may be concluded that reagents do not generate significant wear of the microsystem and polymer presence does not affect the NPs’ formation. The XRD representative diffractogram (Figure 8C) of the synthesized magnetite NPs reveals peaks corresponding to pure crystallites of magnetite with no impurities; the peaks corresponding to the Bragg diffraction planes of the crystalline phase of Fe_3_O_4_ were identified (220, 311, 400, 422, 511, 440) [71,72] and corroborated the absence of precursor impurities and unwanted crystalline phases.

**Figure 10:**
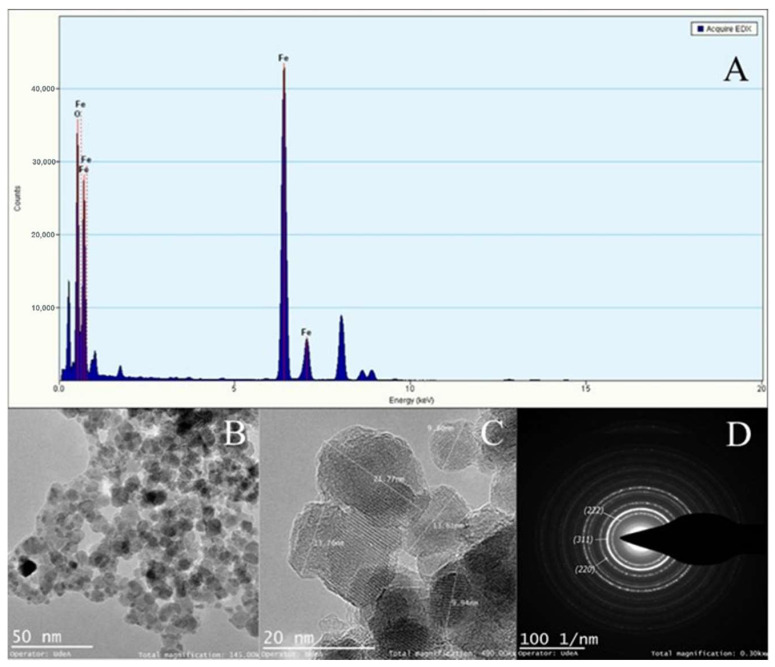
TEM−EDX analysis of magnetite particles manufactured by the TB micromixer. (**A**) Elemental analysis confirmed the abundance of iron. (**B**) Low magnification imaging confirmed the typical morphology of magnetite crystals. (**C)** High magnification imaging allowed the calculation of an average particle diameter of 11.96 ± 4.1 nm. (**D**) Selected area electron diffraction with indexed planes that correspond to magnetite crystalline planes.

**Figure 11:**
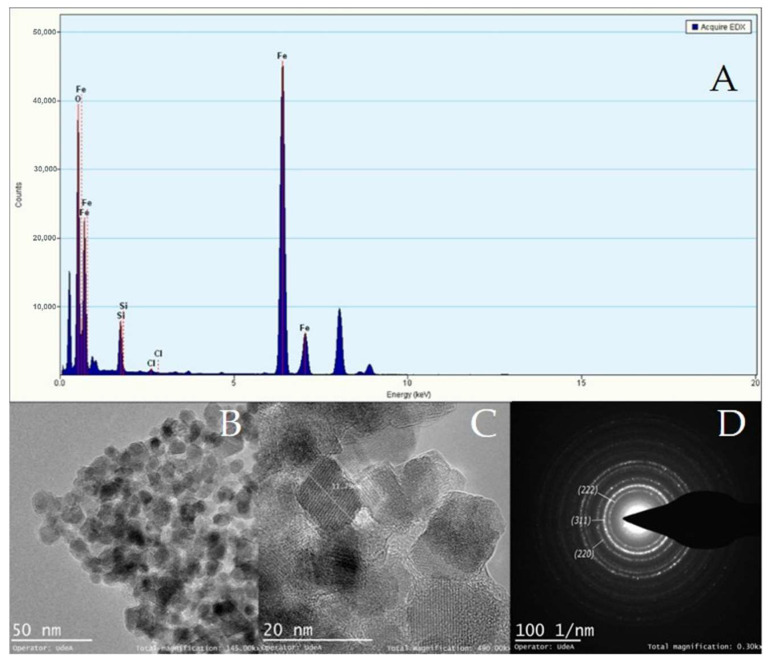
TEM−EDX analysis of magnetite particles manufactured by the 3DB micromixer. (**A**) Elemental analysis confirmed the abundance of iron. (**B**) Low magnification imaging confirmed the typical morphology of magnetite crystals. (**C**) High magnification imaging allowed the calculation of an average particle diameter of 12.70 ± 2.8 nm. (**D**) Selected area electron diffraction with indexed planes that correspond to magnetite crystalline planes.

Figure 9, Figure 10 and Figure 11 show the TEM micrographs, EDX, and selected area electron diffraction (SAED) analyses for magnetite NPs manufactured with the CB micromixer (Figure 9), the TB micromixer (Figure 10), and finally, the 3DB micromixer (Figure 11). The mean particle diameters for the Fe_3_O_4_ NPs synthesized by the SB, TB, and 3DB micromixers were 10.14 ± 2.8 nm, 11.96 ± 4.1 nm, and 12.70 ± 2.8 nm, respectively. The SAED analyses revealed the presence of magnetite’s crystalline planes for all the samples [73].

### 3.4. Water and Energy Consumption Results

**Figure 12:**
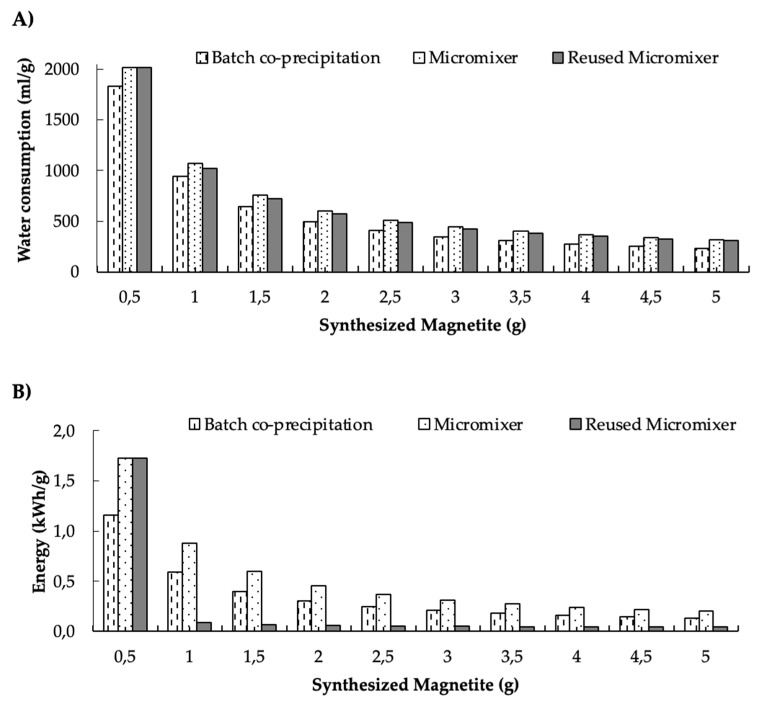
Comparison of consumption between co−precipitation, micromixer−based method, and micromixer reuse methods for the synthesis of magnetite nanoparticles (NPs): (**A**) water consumption per NPs’ gram (mL/g), (**B**) energy consumption per NPs’ gram (kWh/g).

The 3DB micromixer was selected for the water and energy consumption analysis because it requires multiple PMMA layers for its manufacture, a procedure that has proved to lead to higher energy consumption [74]. Therefore, the information presented in this section focuses on the 3DB micromixer as it represents the least favorable energy consumption case of all the micromixers. The water and energy consumption for the synthesis of 0.5 g of magnetite nanoparticles through the two synthesis methods (i.e., conventional batch and with the assistance of micromixers) are detailed in Figure 12. Additionally, we included the data for a reused micromixer to evaluate whether a potential reduction in water and energy consumption are achievable through this approach. The data are presented as a function of the produced mass of NPs. The results indicate that water consumption for the micromixers is slightly higher than for co-precipitation and reusing them for the synthesis (Figure 12A). This is most likely due to the water consumed for the cooling-down process after the micromixer’s PMMA layers were glued together by heating them up. The energy consumption of the micromixers was also higher than the other two synthesis methods (Figure 12B).

The significant reduction in energy consumption for the reused micromixer can be explained by it not needing to pass through the laser cutting step (which is the largest contributor to consumption) from the second use onwards. However, there are marked differences in the energy consumption between micromixers, due to the number of PPMA layers involved for laser cutting and subsequent assembly. This energy consumption was 60 times higher than that required for other stages in the synthesis process. In this regard, the TB and SB mixers consist of two PMMA layers, involving an energy consumption of about 0.2 kWh, while the 3DB mixer requires one more layer for assembly, which translates into nearly 0.3 kWh in energy consumption for laser cutting.

As the micromixers are reused, the water consumption steeply decreases by nearly 50% for the second synthesis, and then for the third onwards, it declines gradually by about 14% per cycle. A similar trend was also observed in the case of energy consumption, with an initial decrease of 95% followed by almost insignificant reductions from the third cycle onwards. There is also a clear reduction in water and energy consumption as the amount of synthesized NPs increases. This result is very attractive from the processing viewpoint, as it makes it feasible to conceptually design and test scaling-up strategies that involve coupling of multiple micromixers, operating continuously. However, future work should be dedicated to evaluating yield, possible changes in physicochemical properties, and the potential environmental impacts of different micromixer coupling strategies for production at a larger scale.

## 4. Discussion

According to the results of our CFD simulations, the three designed micromixers can develop the mixing patterns required to produce magnetite nanoparticles. However, the yield performance of the SB micromixer is likely to be compromised by the presence of large dead zones along the microchannels (Figure 6), which may lead to uncontrolled nucleation and growth processes. This, in turn, might be detrimental to maintaining homogeneous particle size distribution, and uniform morphologies and crystalline structures [75]. This poor mixing behavior has been previously observed in microfluidic devices that incorporate channel geometries such as sinusoidal, T-shaped, and squared. In contrast, the high shear rate levels achieved by the 3DB micromixer provide opportunities for superior mixing patterns (Figure 7), which led to smaller and more uniform particle size distribution compared with the other two micromixers (Figure 8). Previous studies have shown that some microreactors for nanoparticle synthesis are able to produce homogeneous particle size distributions and to allow higher reproducibility compared to bulk synthesis schemes [76,77,78,79,80,81]. Such reports demonstrated that the prevalent geometries to achieve such high performance are herringbone micromixers, coil, and microcapsules [82]. In general, in addition to providing high shear rates, such systems allow better control of process variables such as temperature, flow rates, residence times, and reagents ratios [79,80,81]. From the mechanistic viewpoint, recent studies have provided additional evidence that correlated the success of these synthesis devices with the control of advection, nucleation, and growth processes [64,82,83]. This high level of control over such phenomena has been thought to directly impact the nucleation and subsequent growth of the nanoparticles. In this regard, the TEM micrographs (Figure 9, Figure 10 and Figure 11) confirmed similar morphologies for the magnetite NPs obtained with the three devices. Moreover, similar crystalline structures were observed for all the obtained materials, as evidenced by the collected electron diffraction patterns. Finally, the EDX showed the presence of insignificant traces of Silicon and Chloride contaminants in the samples, which appeared to have no impact on the crystalline structure and morphology of the NPs. Although conventional methods such as co-precipitation [84,85,86,87,88,89] can lead to a sharper NP size distribution, continuous synthesis proved to be well-suited to allowing the control of specific parameters such as the shear rate, concentration of reagents, reaction time, and temperature, which all interplay to determine the reaction performance and energy consumption of the process. The novelty of the current work lies in the easy low-cost manufacture method both for the 2D micromixers and the 3D micromixer; especially for 3D systems, low-cost manufacture tends to be more complex, involving advanced techniques [88]. 

The results showed that energy consumption was about 33% higher than that required for the conventional process, but it decreased considerably as the amount of NPs produced is increased. Recent studies have reported that it is possible to reduce impacts on the environment and human health by considering alternative synthesis schemes based on eco-friendly methods [89]. For example, Marimon-Bolivar et al. [54] showed that the energy spend in the production of magnetite nanoparticles, starting from common raw materials such as iron (II) and ammonium hydroxide, led to an impact 10 times greater than green synthesis schemes. This can be explained by the high impact of such raw materials on various categories such as global warming, human toxicity, photochemical ozone formation, and resource depletion.

Our NP synthesis method based on the 3DB micromixer system led to a reduction in energy consumption of about 76% compared to the traditional batch co-precipitation method, when the system is sequentially reused for the synthesis of additional NPs. This can be explained by not needing to consume any new PMMA for the manufacturing of new devices between syntheses. In this regard, it might be worth replacing such PMMA with recycled PMMA.

Regarding water consumption by the 3DB micromixer, the results showed about a 17% increase compared to the conventional batch co-precipitation method. This water loss can be attributed to leak testing and the refrigerant needed to cool down the micromixer after gluing the PMMA layers during the manufacturing stage. In the LCA study by Bartolozzi et al. [89], the water consumption associated with the production of nanostructured materials, for water remediation purposes, contributed about 10% of the water resource depletion category. However, compared with the use of other raw materials, water use can contribute to an almost 100% impact reduction on this category. As for the energy consumption, when the micromixer is reused for the synthesis, there is a reduction in water consumption that approached 4%.

## 5. Conclusions

Magnetite nanoparticles (MNPs) have attracted significant attention due to their unique properties and numerous applications in several fields, ranging from nanomedicine to remediation of industrial wastewaters. This has led to a projected increase in their global demand over the next few years. For this reason, there is an imperative need to define routes for their production at a large scale, under stringent quality control parameters based on the consistency of key physicochemical properties such as particle size distribution, crystallinity, and morphological features. Thus far, the simplest and most inexpensive synthesis method is based on the co-precipitation of iron chlorides but, depending on how close the conditions are to ideal mixing, the properties of the obtained materials might vary significantly. This is due to changes in the shear rate, which in turn, largely control the nucleation and growth processes responsible for the final product properties. Here, we proposed to address these challenges by a scaling-down approach for continuous production aided by micromixing devices, to take advantage of their unique fluid dynamics where mixing processes are highly controlled. To accomplish this, we designed and evaluated three micromixing devices in silico via multiphysics simulations; two of the micromixers had 2D microchannel configurations and a third one had a 3D arrangement, with abrupt changes in fluid direction. The simulations showed low shear rate values for the 2D devices and the presence of dead volumes at sharp edges within the microchannels’ paths. In the case of the 3D micromixer, the calculated shear rates were the highest but there were also dead volume zones. A further calculation of the concentration profiles expected for magnetite within the devices revealed quite close final values for all of them. Importantly, it appears that such concentrations are achieved more rapidly by the 3D device. Testing the devices experimentally in the MNPs’ synthesis supported these notions, as evidenced by the lowest particle diameter and sharpest particle size distribution being obtained with the 3D device. Despite the differences in velocity, shear rate, and MNPs’ concentration profiles, all the devices led to MNPs with typical morphologies and crystalline structures.

To evaluate whether the 3D device was potentially useful for a large-scale application, we conducted an LCA inventory to estimate water and energy consumption as basic indicators of the possible environmental impact associated with its operation. In comparison with the conventional process, the synthesis assisted with the micromixer led to an average increase in energy consumption of about 33% and in water consumption of 17% on average. When the micromixers are reused, the consumption of energy and water were reduced by about 84% and 4%, respectively. This suggests that a possible avenue to improve the environmental indicators of the device is by manufacturing it with recycled PMMA. Moreover, experimental tests to measure the performance of multiple devices operating in series and/or parallel arrangements are of paramount importance to make more sound estimates regarding the overall sustainability of a real large-scale operation. Taken together, our results provide robust evidence of the potential of micromixing devices to produce high quality MNPs according to standards in particle size distribution, crystalline structure, and morphology. Furthermore, this approach appears to rival conventional synthesis schemes’ productivity, mainly due to the possibility of a continuous operation.

## Figures and Tables

**Table 1:** Parameters of reaction simulations.

Parameters	Value	Units
Density of the fluid	1000	kg/m^3^
Viscosity of the fluid	1	mPa.s
Rate constant	1 × 10^−24^	m^30^/(s·mol^10^)
FeCl_2_ molar mass	0.199	kg/mol
FeCl_3_ molar mass	0.270	kg/mol
NaOH molar mass	0.040	kg/mol
Fe_3_O_4_ molar mass	0.232	kg/mol
NaCl molar mass	0.058	kg/mol
H_2_O molar mass	0.018	kg/mol
FeCl_2_ inflow concentration	100	mM
FeCl_3_ inflow concentration	200	mM
NaOH inflow concentration	800	mM
Central inlet normal inflow rate	1	ml/min
Lateral inlet normal inflow rate	0.5	ml/min

**Table 2:** Inventory report of co-precipitation and micromixer-based synthesis methods.

Method	Inventory	Amount	Unit
Co-precipitation	**Inputs**		
	*Reagents preparation*		
	Iron (II) chloride tetrahydrate $({\mathrm{FeCl}}_{2}$)	0.43	g
	Iron (III) chloride hexahydrate $({\mathrm{FeCl}}_{3}$)	1.17	g
	Sodium hydroxide (NaOH)	0.69	g
	Tetramethylammonium hydroxide (TMAH)	0.0012	L
	Energy (Precision scale)	0.0033	kWh
	Water consumption	0.026	L
	Energy (Stirring plate)	0.0343	kWh
	*Synthesis*		
	Energy (Syringe pump)	0.01067	kWh
	Energy (Stirring plate)	0.5150	kWh
	*Washing and re-dispersion*		
	Water consumption	0.89	L
	Energy (Vortex)	0.0176	kWh
	**Outputs**		
	Wastewater	0.9	L
Micromixer	**Inputs**		
	*Micromixers*		
	Acrylic (PMMA 3 mm)	0.000039375	m^3^
	Energy (Laser cut)	0.7	kWh
	Ethanol 70%	10	mL
	Ethanol 96%	5	mL
	Energy (hot plate)	0.09	kWh
	Acrylic glue	1.5	g
	Water consumption	0.053	L
	*Reagents preparation*		
	Iron (II) chloride tetrahydrate $({\mathrm{FeCl}}_{2}$)	0.43	g
	Iron (III) chloride hexahydrate $({\mathrm{FeCl}}_{3}$)	1.17	g
	Sodium hydroxide (NaOH)	0.69	g
	Tetramethylammonium hydroxide (TMAH)	1	mL
	Energy (Precision scale)	0.005	kWh
	Water consumption	0.06579	L
	Energy (Stirring plate)	0.0343	kWh
	*Synthesis*		
	Energy (Syringe pump)	0.0072	kWh
	*Washing and re-dispersion*		
	Water consumption	0.89	L
	Energy (Vortex)	0.0176	kWh
	**Outputs**		
	Water consumption	0.0525	L
	Wastewater	0.9	L

## Data Availability

The data presented in this study are available on request from the corresponding author.

## Supplementary Materials

The following are available online at https://www.mdpi.com/article/10.3390/mi13060970/s1, Figure S1: Mesh convergence analysis for the Serpentine-based mixer, Figure S2: Mesh convergence analysis for the Triangular-based mixer, Figure S3: Mesh convergence analysis for 3D-based mixer.
