# Supplementary material for: CFD Analysis and Life Cycle Assessment of Continuous Synthesis of Magnetite Nanoparticles Using 2D and 3D Micromixers
Source: Micromachines (Basel). 2022 Jun 19;13(6):970. doi: 10.3390/mi13060970 (PMC9230433; doi:10.3390/mi13060970)
Supplement: Supplementary file 1 [file micromachines-13-00970-s001.zip › micromachines-1705621-supplementary.pdf]

Supplementary Information

2D and 3D micromixers for the synthesis of magnetite nanoparticles: CFD analysis and life cycle assessment

Sergio Leonardo Florez [1], Ana Lucia Campaña [1], M. Juliana Noguera [1], Valentina Quezada [2], Olga P. Fuentes [1], Juan C. Cruz [2] and Johann F. Osma [1,*]

[1] Department of Electrical and Electronic Engineering, Universidad de los Andes, Cra. 1E No. 19a-40,
Bogota 111711, Colombia; sl.florez10@uniandes.edu.co (S.L.F.); al.campana10@uniandes.edu.co (A.L.C.); mj.noguera10@uniadnes.edu.co (M.J.N.); op.fuentes@uniandes.edu.co (O.P.F.)

[2] Department of Biomedical Engineering, Universidad de Los Andes, Cra. 1E No. 19a-40, Bogota 111711, Colombia; v.quezada@uniandes.edu.co (V.Q.); jc.cruz@uniandes.edu.co (J.C.C.)

* Correspondence: jf.osma43@uniandes.edu.co; Tel.: +57-1-339-4949

Supplementary Figures

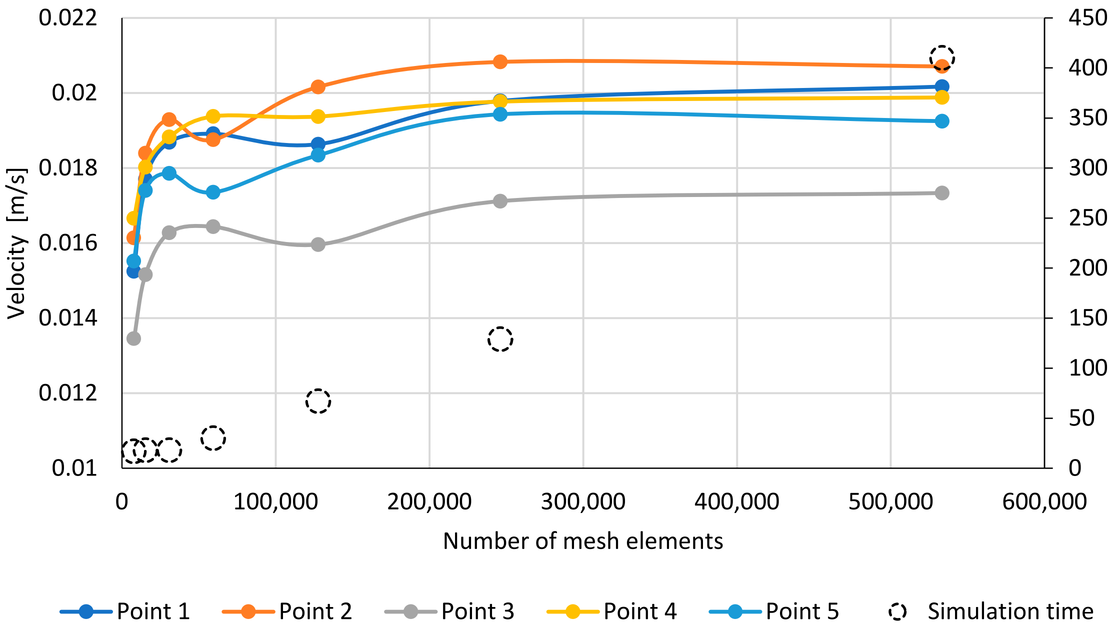

Figure S1. *Mesh Convergence analysis for the Serpentine-based mixer.*

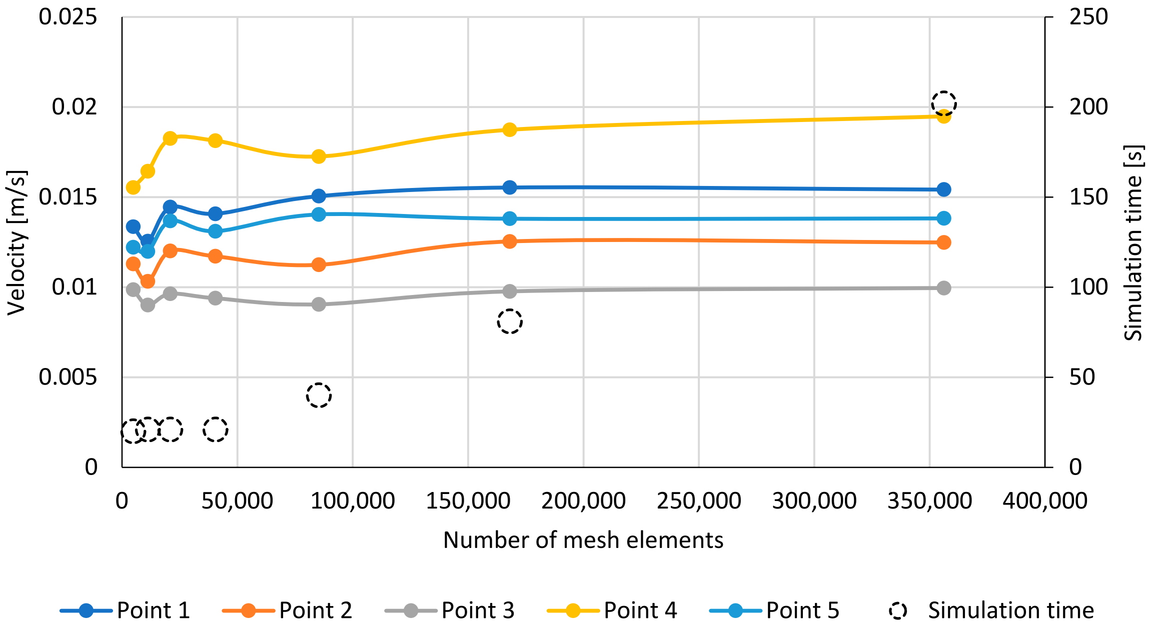

Figure S2. *Mesh convergence analysis for the Triangular-based mixer.*

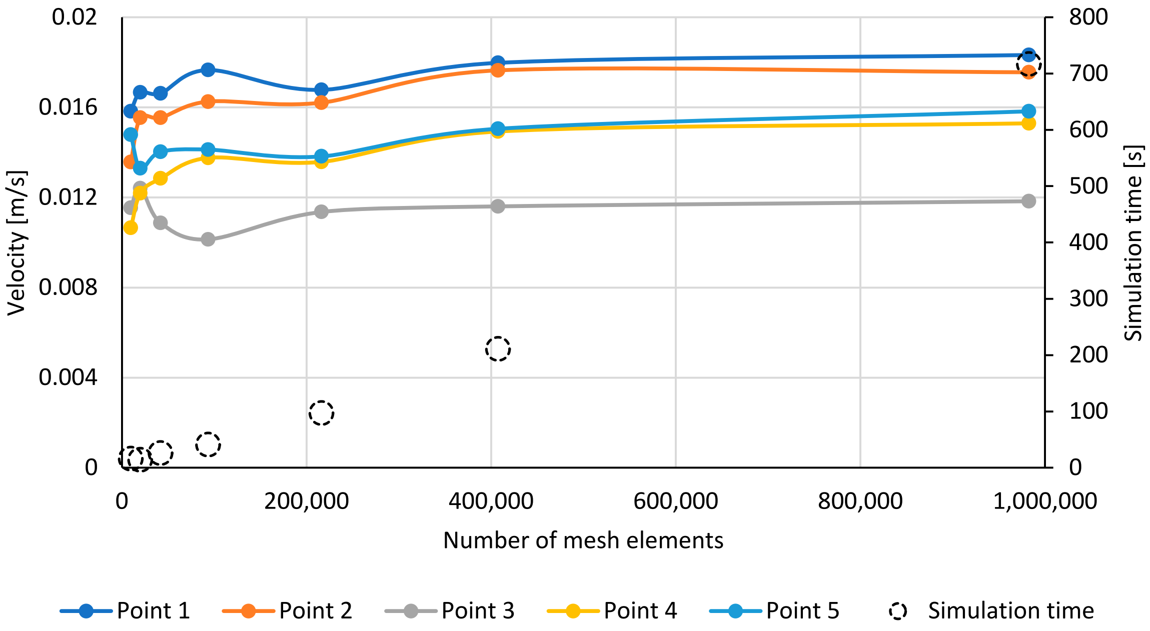

Figure S3. *Mesh Convergence analysis for 3D-based mixer.*
